# Effects of Dietary Fat to Carbohydrate Ratio on Obesity Risk Depending on Genotypes of Circadian Genes

**DOI:** 10.3390/nu14030478

**Published:** 2022-01-22

**Authors:** Jinyoung Shon, Yerim Han, Yoon Jung Park

**Affiliations:** 1 Department of Nutritional Science and Food Management, Ewha Womans University, Seoul 03760, Korea; shon.jinyoung.layla@gmail.com (J.S.); hanyelim97@naver.com (Y.H.); 2 Graduate Program in System Health Science & Engineering, Ewha Womans University, Seoul 03760, Korea

**Keywords:** macronutrient distribution, circadian gene, genetic variant, single nucleotide polymorphisms (SNPs), obesity

## Abstract

Although the impacts of macronutrients and the circadian clock on obesity have been reported, the interactions between macronutrient distribution and circadian genes are unclear. The aim of this study was to explore macronutrient intake patterns in the Korean population and associations between the patterns and circadian gene variants and obesity. After applying the criteria, 5343 subjects (51.6% male, mean age 49.4 ± 7.3 years) from the Korean Genome and Epidemiology Study data and nine variants in seven circadian genes were analyzed. We defined macronutrient intake patterns by tertiles of the fat to carbohydrate ratio (FC). The very low FC (VLFC) was associated with a higher risk of obesity than the optimal FC (OFC). After stratification by the genotypes of nine variants, the obesity risk according to the patterns differed by the variants. In the female VLFC, the major homozygous allele of *CLOCK* rs11932595 and *CRY1* rs3741892 had a higher abdominal obesity risk than those in the OFC. The GG genotype of *PER2* rs2304672 in the VLFC showed greater risks for obesity and abdominal obesity. In conclusion, these findings suggest that macronutrient intake patterns were associated with obesity susceptibility, and the associations were different depending on the circadian clock genotypes of the *CLOCK*, *PER2*, and *CRY1* loci.

## 1. Introduction

The circadian clock governs 24 h rhythms and regulates the sleep–wake cycle. In mammals, circadian rhythms influence metabolism and physiological processes [1]. Furthermore, the circadian clock regulates glucose and fat metabolism and energy metabolism by coordinating the expression of clock-controlled genes [1,2]. The circadian core genes, including the circadian locomotor output cycle kaput (*CLOCK*), aryl hydrocarbon receptor nuclear translocator-like (*ARNTL*, also known as *BMAL1*), period homolog (*PER1*, *PER2*), and cryptochrome (*CRY1*, *CRY2*) regulate the circadian rhythm mechanism [1,3]. The ARNTL-CLOCK complex drives the transcription of *PER* and *CRY* genes by binding to enhancer elements. Increased proteins of PER and CRY inhibit ARNTL-CLOCK-mediated transcription. This transcription–translation negative feedback loop leads the circadian rhythm, which takes 24 h [3,4].

Multiple evidence from mouse models and human studies have reported a link between the risk of disease and clock genes [5,6,7,8,9,10,11,12,13,14]. Moreover, genetic variations of clock genes might play a role in metabolic disorders. Single nucleotide polymorphisms (SNPs) of *CLOCK* and *ARNTL* influence body weight control, the development of obesity, and susceptibility to metabolic diseases [12,13,15,16,17,18,19,20]. Additionally, the SNPs of circadian genes are associated with eating behavior and dietary intake, including carbohydrate, protein, and fat, and this association contributes to the modulation of physiological responses [21,22,23,24,25].

The master clock located in the hypothalamic suprachiasmatic nucleus can be regulated by the light–dark cycle [1,26,27], whereas peripheral clocks in peripheral tissues, such as the liver and heart, are entrained by other environmental factors [1,6,26]. Dietary nutrients are a crucial driver for oscillation of the peripheral circadian clock [28,29]. Several studies have reported an altered phase of the peripheral clock under time-restricted feeding conditions or high-fat diet feeding experiments [30,31,32]. Feeding mice with a high-fat diet induced reprogramming of the liver clock and changes in eating behavior [30,33,34]. Furthermore, substitution of a diet component with another component influenced phase shifts in the liver circadian clock [35]. The ketogenic diet, which comprises high-fat with low-carbohydrate and protein contents, affected the peripheral circadian clocks and drive tissue-specific oscillation of clock-controlled genes [36]. A low-carbohydrate and high-protein diet altered the expressions of circadian genes and key gluconeogenic regulatory genes, resulting in mild hypoglycemia [37]. These results indicate that dietary macronutrient composition is a strong factor for the regulation of peripheral clocks and clock-controlled genes involved in metabolic processes.

Dietary macronutrients are important to maintain health and physiological functions. In previous nutritional intervention studies, the results mainly focused on the effects of low-fat or low-carbohydrate diets on obesity-related features such as weight control [38,39,40,41]. However, most interventional diets that modify macronutrient distribution are based on an energy deficit or investigated over the short term, resulting in inconsistent metabolic outcomes. One of the most interesting studies carried out by Solon-Biet et al. investigated the effects of macronutritional challenges using a chronic ad libitum-fed mouse model [42]. Interestingly, a ‘high-protein and low-carbohydrate diet’ induced negative outcomes related to metabolic health and longevity. In contrast, a ‘low-protein and high-carbohydrate diet’ improved health and extended the lifespan. This suggests that results derived from dietary interventions are not consistent with actual responses under a long-term diet without calorie restriction. Moreover, given that the distributions of dietary macronutrients differ between populations, results from western-style intervention diets (e.g., low-protein and high-fat diet and low-carbohydrate diet) are hard to apply to Asian populations. Thus, the understanding of dietary macronutrient distribution must be considered in the context of population health improvement.

Several studies that investigated the effects of nutritional challenges on the circadian system reported that altered feeding cycles under an obesogenic diet were related to metabolic disorder [43,44]. Macronutrient intake and the timing of the caloric intake were related to the sleep cycle and influence of obesity risk [45,46]. Moreover, circadian clock gene SNPs and energy and fat intake were associated with metabolic health and obesity-related outcomes [23,24,25]. Collectively, these results suggest that dietary macronutrient intake and circadian genes contribute to susceptibility to metabolic diseases. However, the potential role of circadian gene SNPs and dietary macronutrient distribution was not investigated for its link to disease risk. Therefore, in this study, we defined Korean macronutrient intake patterns and analyzed the effects of an association between patterns and circadian clock gene variants and obesity risk.

## 2. Materials and Methods

### 2.1. Study Data and Subjects

**Figure 1:**
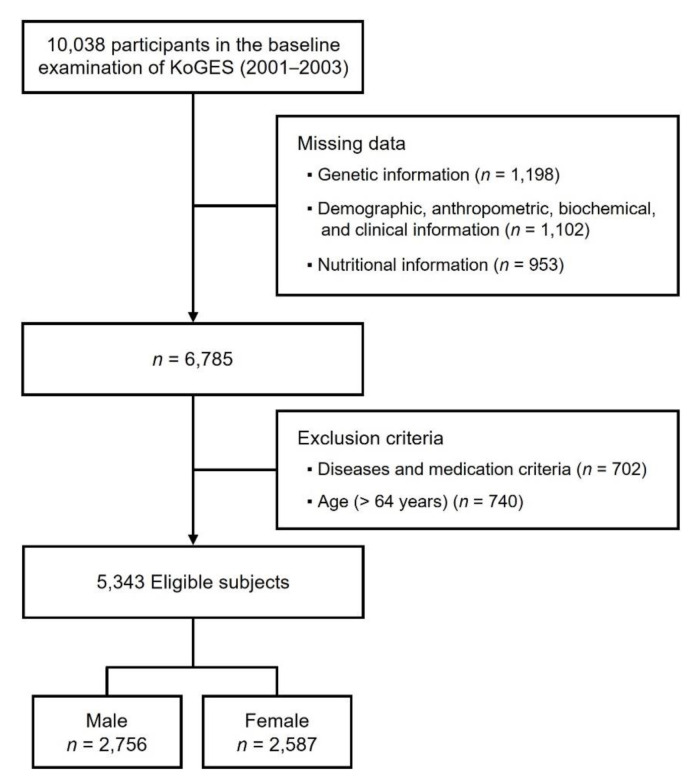
A flow chart of the study population.

This study used the Korean population data from the Korean Genome and Epidemiology Study (KoGES), provided by the Center for Genome Science, National Institute of Health, Korean Centers for Disease Control (KCDC) and Prevention, Chungcheongbuk-do, Korea [47]. A local community-based cohort was obtained from urban (Ansan) and rural (Ansung) regions, containing genomic, demographic, anthropometric, biochemical, clinical, and nutritional information. All participants provided written informed consent, and cohort data were surveyed every 2 years on a follow-up basis since 2001. We used the baseline examination dataset for this study. Among 10,038 subjects, 3253 were excluded due to missing data (Figure 1). Exclusion criteria (cancer, dementia, stroke, steroid drugs, insulin therapy, oral diabetes medication, thyroid drugs, and hormone replacement therapy) were applied for the elimination of effects derived from diseases and drugs on food intake. Finally, we investigated 5343 subjects aged 40~64 years, of which 2756 were male (mean age 48.9 ± 7.0 years), and 2587 were female (mean age 49.9 ± 7.6 years). The study was approved by the Institutional Review Board of Ewha Womans University, Seoul, Korea (IRB approval number: ewha-202105-0003-01).

### 2.2. Selection and Analysis of SNPs

**Figure 2:**
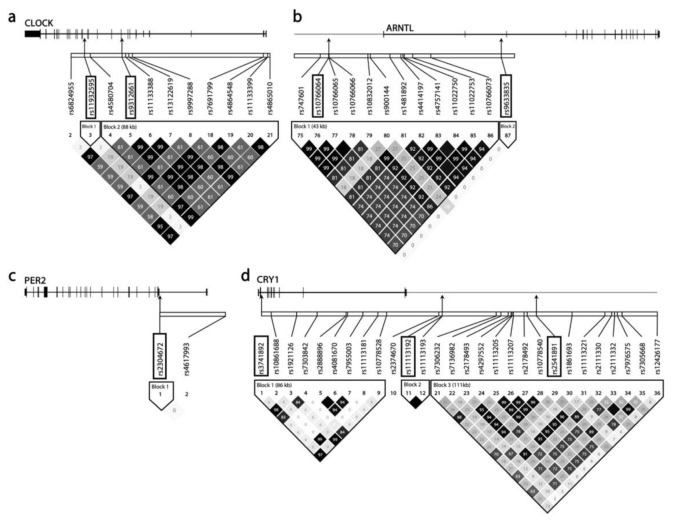
Pairwise linkage disequilibrium (LD) blocks for SNPs of the circadian gene locus. The horizontal white bar depicts DNA segmentation of circadian gene locus, *CLOCK* (**a**), *ARNTL* (**b**), *PER2* (**c**), and *CRY1* (**d**). Each diamond represents the magnitude of LD for a single pair of markers. The numbers inside the diamonds indicate the r^2^ value. The blocks are shaded corresponding to the r^2^ from no LD (white, r^2^ = 0) to strong LD (black, r^2^ = 1.0), and gray tones indicate intermediate. A part of SNPs included data was shown, and the black arrows indicate SNPs analyzed in this study.

Genomic DNA derived from blood samples was genotyped with the Affymetrix Genome-Wide Human SNP Array 5.0 kit (Affymetrix, Inc., Santa Clara, CA, USA) [48], and 1000 genome sequences were used for imputation [49]. After applying the Bayesian Robust Linear Modeling with Mahalanobis Distance (BRLMM) algorithm and standard quality control procedures, samples with a missing call rate >4%, heterozygosity >30%, gender incompatibility, or obtained from subjects who had cancer were excluded [50]. Among 352,228 SNPs, we selected 235 SNPs that were located in the loci of the circadian core genes *CLOCK*, *ARNTL*, *PER1*, *PER2*, *PER3*, *CRY1*, and *CRY2* (Figure 2). SNPs with a high missing genotype call rate (>5%), low minor allele frequency (MAF < 0.05), and low Hardy–Weinberg equilibrium (*p* value < 1 × 10^−6^) were excluded. We conducted linkage disequilibrium (LD)-based pruning (r^2^ > 0.2); one SNP which had the highest MAF was selected from each LD block using PLINK software version 1.09 [51] and Haploview software version 4.1 (Broad Institute of MIT and Harvard, Cambridge, MA, USA) [52]. Utilizing the multitissue expression quantitative loci (eQTL) analysis from the Genotype Tissue Expression (GTEx) projects (release version 8) [53,54], we selected 9 SNPs related to circadian gene regulation (Table 1 and Table A1, Figure 2). A recessive model was used for further investigation due to the small number of subjects of homozygous for the minor allele.

### 2.3. Macronutrient Patterns

A validated semi-quantitative food frequency questionnaire with 103 food items was used for assessing dietary data [55]. The consumption frequency and portion size of items during the previous year were investigated. The sum of the nutrient intake from each food item was calculated to evaluate the average daily energy intake and nutrient intake of each individual. Macronutrient (carbohydrate, fat, and protein) intake was presented as the percentage of total energy intake. Given the protein intake was positively correlated with fat intake in this cohort population (data not shown), we defined fat to carbohydrate ratio (FC ratio) by dividing ‘% energy from fat’ by ‘% energy from carbohydrate’. Subsequently, subjects were categorized by tertiles of the FC ratio: Very low FC (VLFC; the first tertile), Low FC (LFC; the second tertile), and Optimal FC (OFC; the third tertile).

### 2.4. Definitions of the Obesity and Abdominal Obesity

Anthropometric measurements were obtained (i.e., height, weight, waist circumference) by trained staff in cohort study [47]. In the present study, obesity was defined as a BMI ≥ 25 kg/m^2^ according to Asia–Pacific BMI cut-off from the World Health Organization Report [56]. The abdominal obesity was defined as a waist circumference ≥90 cm for males and ≥85 cm for females according to the diagnostic criteria for Korea [57].

### 2.5. Statistical Analysis

Data were presented as the mean ± standard deviation, number, and percentage. ANOVA analysis with Tukey post hoc comparison test was used to identify group differences, and Welch’s ANOVA with Games–Howell test was used to adjust for unequal variances. The Chi-square test was used to analyze categorical variables. Multiple logistic regression analysis was used for exploring the associations between genotypes and disease after adjustment for covariates, such as age, body mass index (BMI), sleep duration, alcohol intake, tobacco consumption, physical activity, energy intake, and number of regular meals. Statistical analyses were performed using SAS software version 9.4 (SAS Institute, Inc., Cary, NC, USA) and RStudio ver.1.2.1335 (RStudio Inc., Boston, MA, USA). A *p*-value of <0.05 was considered to be statistically significant. Bonferroni correction was applied to correct for multiple testing (Bonferroni corrected *p* < 0.011).

## 3. Results

### 3.1. General Characteristics and Nutritional Intake

The main characteristics of all the included participants are shown in Appendix B. After dividing subjects into tertiles of the FC ratio, the general characteristics according to groups were analyzed (Table 2). Subjects in the VLFC group (T1) were older than the LFC group (T2) and the OFC group (T3) (male VLFC: 50.8 ± 7.3 years, LFC: 48.6 ± 6.7 years, and OFC: 47.3 ± 6.4 years; female VLFC: 53.2 ± 7.5 years, LFC: 49.7 ± 7.5 years, and OFC: 46.9 ± 6.3 years). The VLFC showed had a lower BMI than other groups in males (24.7 ± 2.9 kg/m^2^), whereas female VLFC had a higher BMI (25.3 ± 3.4 kg/m^2^). In the VLFC group, the portion of rural subjects was greater than other groups (male VLFC: 43.2% and female VLFC: 58.2%). The proportion of urban subjects was highest in the OFC group (male OFC: 84.8% and female OFC 78.0%). The VLFC had a lower lean body mass and body fat than other groups in males (52.7 ± 5.8 kg and 15.1 ± 4.7 kg, respectively). In contrast, female VLFC had a lower lean body mass (39.8 ± 4.6 kg) and higher body fat (15.7 ± 4.9 kg). Furthermore, the female VLFC showed a higher waist to hip ratio (0.91 ± 0.05) compared to other groups.

The nutritional intake including total energy, carbohydrate, protein, and fat was highest in the OFC group and lowest in the VLFC group. However, carbohydrate intake did not differ by FC group in females. The VLFC group had a significantly higher % of energy from carbohydrate intake (78.2 ± 3.0% in females) and consequently a lower % of energy from protein and fat (11.7 ± 1.4% and 8.6 ± 2.0% in females, respectively) than in other groups (Appendix C). Considering that the Korean Acceptable Macronutrient Distribution Range (AMDR) for carbohydrate is 55~65%, for protein is 7~20%, and for fat is 15~30% of the energy intake for adults [58], the OFC group’s proportion fitted the Korean AMDR.

In contrast, the VLFC and LFC had an inadequate composition of macronutrients, which fell outside the AMDR with a higher carbohydrate and lower fat intake. Because the OFC had a macronutrionally balanced diet with optimal proportions, we designated the OFC as the reference group in our further analysis. The FC ratio was 0.14 ± 0.03, 0.23 ± 0.02, and 0.34 ± 0.08 for male VLFC, LFC, and OFC respectively; and 0.11 ± 0.03, 0.19 ± 0.02, and 0.31 ± 0.09 for female VLFC, LFC, and OFC respectively.

### 3.2. Risk of Obesity by Macronutrient Intake Patterns

The prevalence of disease according to the tertiles of the FC ratio is shown in Table 3. In males, the LFC group had an increased risk of obesity (odds ratio (OR): 1.29, 95% confidence interval (CI): 1.07–1.57) compared with the OFC group. There was no effect of patterns on the incidence of abdominal obesity in males. Interestingly, in females, the VLFC group showed greater odds of obesity and abdominal obesity than in the OFC group (OR: 1.50, 95% CI:1.20–1.86; OR: 1.84, 95% CI 1.36–2.48, respectively).

### 3.3. Macronutrient Intake Patterns, Genetic Variants, and Risk of Obesity

To investigate the association of macronutrient composition and genetic variations of circadian clock genes, we stratified subjects by the genotypes of nine SNPs and analyzed the risk of obesity (Table 4 and Table 5). The homozygous major allele of each SNP in the OFC was used as the reference group in the regression analysis, and the Bonferroni adjustment was used for multiple testing correction.

The risk of disease was increased in the VLFC group, particularly in females (Table 5). In the male VLFC group, the minor allele carriers of *CLOCK* rs9312661, *CRY2* rs7951225, and the GG genotype of *CRY1* rs11113192 showed increased risks of obesity; however, significances were diminished after the Bonferroni correction (Table 4). An interaction between *CRY1* rs11113192 and the FC on obesity was observed (*p*-interaction = 0.009); however, the significance disappeared after multiple corrections. No statistically significant differences were found for abdominal obesity.

In females, both genotypes of *CLOCK* rs9312661 in the VLFC showed an increased incidence of abdominal obesity compared with the reference group (AA genotype, OR: 2.26, 95% CI: 1.43–3.56, *p* = 0.0005; GA/GG genotype, OR: 2.11, 95% CI: 1.38–3.23, *p* = 0.0005). In addition, under the VLFC condition, *CRY1* rs3741892 had a significantly greater obesity risk than the reference regardless of genotype (GG genotype, OR: 1.60, 95% CI: 1.22–2.10, *p* = 0.0007; GA/GG genotype, OR: 1.76, 95% CI: 1.30–2.38, *p* = 0.0002). Intriguingly, the associations between macronutrient intake patterns and obesity risks were different depending on the genotypes of *CLOCK* rs11932595, *PER2* 2304672, and *CRY1* rs3741892. The major allele homozygous, AA genotype, of rs11932595 in the VLFC had a higher risk of abdominal obesity than the reference group (OR: 1.84, 95% CI: 1.32–2.56, *p* = 0.0003), but not in subjects carrying the minor G allele. Regarding *CRY1* rs3741892, which showed a higher obesity risk in both genotypes, the GG genotype, but not the CG/CC genotype, had a greater incidence of abdominal obesity (OR: 1.90, 95% CI: 1.30–2.76, *p* = 0.0008). Moreover, females with the GG genotype of *PER2* rs2304672 in the VLFC had significantly higher risks of obesity and abdominal obesity compared with the references (OR: 1.49, 95% CI:1.18–1.87, *p* = 0.0007; OR: 1.85, 95% CI 1.35–2.54, *p* = 0.0001 respectively), whereas no differences were detected in minor C allele carriers.

### 3.4. Potential Links between Genetic Variants and Gene Regulation

**Figure 3:**
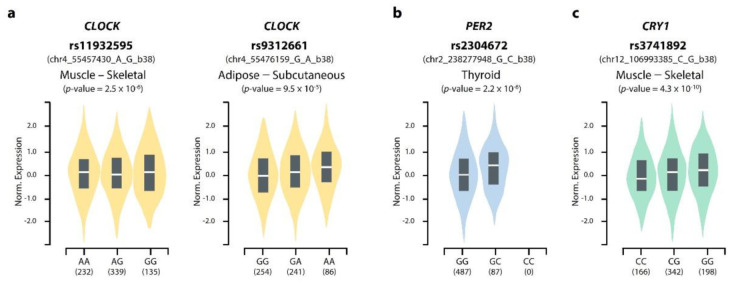
Relationship between genetic variants and circadian gene regulation. Effect of genetic variants on gene expression levels are shown by expression quantitative trait (eQTL) violin plot. The plot indicates the density distribution of samples in each genotype and number of subjects shown under each genotype. The white line in the box plot (black) shows the median value of the expression at each genotype. Association between rs11932595 and rs93126661 with *CLOCK* expression (**a**), Association between rs2304672 with *PER2* expression (**b**), and rs3741892 with *CRY1* expression (**c**). Data analysis was performed using GTEx Portal and included tissue-specific information provided by the website [54].

To explore the potential role of genetic variants on circadian gene regulation, we conducted an eQTL analysis at the SNP selection step. The four SNPs (rs11932595, rs9633835, rs2304672, and rs3741892), which had association with macronutrient intake patterns and obesity risk, contributed to gene expression in various tissues involved in metabolism (Appendix A). For instance, the genotypes of rs11932595 and rs9312661 influence *CLOCK* gene expression in the skeletal muscle, small intestine, colon, pancreas, and subcutaneous adipose tissue (Figure 3). Moreover, thyroidal *PER2* expression is impacted by rs2304672 genotypes, and the *CRY1* expression of the skeletal muscle is affected by rs3741892. Interestingly, the GG genotype of *PER2* rs2304672, which had a significantly increased risk of obesity in our results (Table 4), showed lower expression levels than C carriers (CC genotype: not found in the eQTL violin plot analysis, but a small portion of subjects were present in our data; *n* = 8 males and *n* = 12 females). These findings indicate that genetic variants might influence circadian gene expression levels in important metabolic tissues.

## 4. Discussion

In the present study, we explored macronutrient intake patterns in a Korean midlife population and observed associations between patterns and circadian clock gene variants and obesity. A categorization of the three patterns by the FC ratio revealed the high carbohydrate and relatively low-fat intake of subjects. The prevalence of obesity and abdominal obesity increased in the VLFC compared to the OFC in females. After stratification by the genotypes of nine SNPs, the obesity risk according to the patterns was different according to the genetic variants of *CLOCK*, *PER2*, and *CRY1*. In the VLFC pattern, the major allele homozygous genotype of rs11932595, rs3741892, and rs2304672 had greater risks of obesity and abdominal obesity than the reference group, whereas minor allele carriers had no difference in risk. These findings indicate that macronutrient intake patterns were associated with obesity susceptibility, and the associations were dependent on circadian clock genetic variants, particularly in females. To the best of our knowledge, this is the first study to investigate the roles of dietary macronutrient distribution and circadian clock genes in disease risk in the Korean population.

Dietary macronutrients induced alterations of circadian clock gene expression and phase shift in tissues [30,33,34,35]. The substitution of dietary components induced phase shifts of the hepatic circadian clock [35]. A high-fat diet altered the expression of circadian clock genes in the liver and adipose and, consequently, induced changes in the periods of circadian rhythms with advanced phase [30,32,33]. Mice fed a high-fat diet for 10 weeks revealed the reprogramming of the liver clock through the alternative oscillation of transcripts and metabolites in the liver [34]. The molecular mechanisms of reprogramming induced by high fat are the impairment of CLOCK:BMAL1 chromatin recruitment and a newly oscillating pattern of the peroxisome proliferator-activated receptor gamma (PPARγ), a nuclear receptor involved in glucose and lipid metabolism. The ketogenic diet, which consists of high fats and low carbohydrates, promotes BMAL1 chromatin recruitment in the liver and induces the tissue-specific oscillation of the peroxisome proliferator-activated receptor alpha (PPARα) and its target genes [36]. In a human study, the regulation of dietary fat and carbohydrate content altered the oscillations of peripheral clock genes and inflammatory genes [59]. A high-protein diet affected the expression of circadian genes and key gluconeogenic genes phosphoenolpyruvate carboxykinase (*PEPCK*) and glucose-6-phosphatase (*G6Pase*) in liver and kidney [37]. Therefore, interactions between dietary macronutrient distribution and circadian clock genes might influence downstream clock-controlled genes, leading to changes in metabolic outcomes. In this study, we identified macronutrient intake patterns in a Korean population and observed that the VLFC pattern was associated with increased risks of obesity and abdominal obesity. Moreover, this association was dependent on circadian genetic variants of *CLOCK*, *PER2*, and *CRY1*. Thus, these results suggest that the identification of patterns of dietary macronutrient distribution and understanding the effects of interactions between patterns and circadian genes are essential for the prevention of obesity.

To investigate the potential contribution of genetic variants to gene regulation, we selected nine SNPs by eQTL analysis. The eQTL from the GTEx portal uncovered genetic variants, including SNPs, that influenced differential levels of gene expression [53]. In the GTEx portal, tissue-specific gene expression and SNPs associations were investigated across all 49 human tissues. A combination of eQTL and SNP is useful for the comprehensive exploration of genetic effects on phenotypic variation and disease [60]. One study, which investigated disease-associated SNPs by applying an eQTL analysis, showed that several SNPs regulated gene expression levels in a tissue-specific manner, for example, the IRS1 gene in adipose tissue and influenced the risk of obesity and type 2 diabetes [61]. Rs1801260, a *CLOCK* polymorphism, has a role in the development of obesity, diabetes, and metabolic syndrome [12,18,19,20,23]. In a Korean population study, which used the same cohort data as our research but utilized a different genotype array chip, *CLOCK* rs1801260 affected the incidence of metabolic syndrome, and the association was more apparent after the stratification of monounsaturated fatty acid intake [22]. Moreover, the haplotype of three SNPs (rs1801260–rs11932595–rs4580704) influenced the risks of overweight and hyperglycemia. Considering the eQTL information of rs1801260 and rs11932595 was related to the differential expression of *CLOCK* in various tissues, these results imply that circadian genetic variants might regulate circadian genes as well as clock-controlled genes, resulting in different metabolic phenotypes. Having investigated the effects of genetic variants and macronutrient patterns on obesity risk, we found four significant SNPs. According to the eQTL analysis, the four SNPs influenced gene expression in various tissues (Appendix A). Genetic variants of *CLOCK*, *PER2*, and *CRY1* are associated with gene expression in muscle, adipose, and thyroid, which are known to regulate metabolism. In particular, the rs2304672 genotypes showed differential *PER2* expression levels, which were lower in the GG genotype compared with the GC genotype. *PER2* rs2304672 genetic variants were previously associated with psychiatric disorders including bipolar disorder, depression, and diurnal preference [62,63,64]. Two studies reported that the G allele of rs2304672 had morning preference [64,65], but no significance was found in a young Korean population [66]. In overweight/obese participants on a weight-reduction program, the G allele carriers of rs2304672 showed a lower waist to hip ratio values but had a greater probability of dropping out from the program with constant snacking and skipping breakfast than the CC genotype [21]. Moreover, the interactions between rs2304672 and plasma fatty acids on the modulation of lipoprotein-related biomarkers were reported [67]. Among metabolic syndrome patients with high plasma saturated fatty acid levels, the G allele carriers had higher plasma triglycerides, apolipoprotein C, and apolipoprotein B-48 concentrations than the CC genotype. Given that PER2 also interacts with nuclear receptors including PPARα and can regulate the expression of nuclear receptor target genes involved in lipid metabolism, *PER2* polymorphisms could contribute to metabolic disorder vulnerability [68]. In addition, rs2304672, which is located in the 5′ untranslated region of the *PER2* gene, was suggested to alter the secondary structure of the transcript or change the folding of *PER2* mRNA, resulting in differential translation levels or functionality of proteins between the genotypes [64,67]. Although the mechanisms underlying disease susceptibility is not fully understood, these results support an important role of *PER2* genetic variants on obesity by regulating circadian gene expressions and functions. Further analysis is required to investigate the gene regulatory mechanisms of these SNPs.

We displayed distributions of Korean macronutrient intake patterns by the FC ratio stratification (Appendix C). The notable features in our study were a high proportion of carbohydrate intake and a positive correlation between protein and fat intake. The VLFC group, which had a low fat to carbohydrate ratio, had the highest carbohydrate intake and relatively low intake level of fat and protein. In contrast, the OFC group had a lower carbohydrate intake and increased fat and protein intake than the VLFC. Moreover, the OFC group had a balanced distribution with appropriate proportions of macronutrients that met the Korean AMDR.

The dietary intake proportion differed across populations. Western diets are characterized as having a high dietary level of saturated fats and refined carbohydrates and low levels of fiber. Previous studies have reported the effects of conventional dietary approach which applied a low-carbohydrate or low-fat diet to weight loss and improvement of obesity [38,69]. The types of intervention diets usually suggested for controlling weight can be categorized into three types: low-carbohydrate, low-fat, and moderate macronutrients [38]. Low-carbohydrate diets including Atkins and Zone diets contain 15~40% energy from carbohydrates, 30% energy from proteins, and 30~55% energy from fats. The low-fat diet is composed of 60~70% of energy from carbohydrates, 10~15% from proteins, and 10~20% from fats. In addition, a high-protein, low-fat diet had positive effects on body weight loss and metabolic benefits [69,70,71,72], providing 44%, 31%, and 25% of energy from carbohydrates, proteins, and fats, respectively. These results imply that previously utilized intervention diets are designed for western-style macronutrient distribution. For instance, there is a large difference in distribution between ‘low-carbohydrate diets’ or ‘high-protein and low-fat’ diets and Asian populations who have a much higher carbohydrate intake.

Although accumulating evidence supports the contribution of dietary macronutrient distribution to the development and prevention of metabolic diseases, the relationship between macronutrients and metabolic benefit is still controversial. Several research groups demonstrated that a low-carbohydrate diet is more effective at reducing weight, fat mass, and serum triglycerides and improving metabolic syndrome than a low-fat diet [73,74,75,76,77]. In contrast, other results showed both diets led to similar effects on weight control or clinical markers including glucose level, lipid profile, and blood pressure [40,74]. A meta-analysis study comparing 14 popular dietary programs found that most diets reduced weight and improved blood pressure at 6 months; however, the effects disappeared at 12 months [38]. One issue to consider is that previously conducted intervention diets modifying macronutrient distribution were usually based on energy restriction and have a short-term design. However, there were mouse studies with diets varying in protein to carbohydrate ratio, which examined the interactive effects of dietary macronutrient distribution and metabolic outcomes under *ad libitum* conditions [42,78]. Short-term ‘high-protein and low-carbohydrate’ diets decreased insulin sensitivity, impaired glucose tolerance, and increased triglycerides, resulting in metabolic dysregulation [78]. In contrast, ‘low-protein and high-carbohydrate’ diets prevented adiposity gain and improved metabolic health including insulin, glucose, and lipid levels, despite increased energy intake. As a result of chronic feeding over a lifetime in mice, ‘high-protein and low-carbohydrate’ diets reduced food intake and adiposity; however, they caused negative outcomes in metabolic health and shortened longevity [42]. Long-term ‘low-protein and high-carbohydrate’ diets increased food intake, body weight, and adiposity, but there were positive impacts on health and a longer lifespan, possibly through the regulation of mammalian target of rapamycin (mTORC1) activation [42].

Low-carbohydrate diets replaces carbohydrates with proteins or fats, a typical example is a ketogenic diet. The metabolic benefits of the low carbohydrate diets are inconsistent. Low-carbohydrate diets with increased fat or protein have been reported to be effective for weight loss and improving the lipid profile [39,75,76]. A meta-analysis comparing ‘low-carbohydrate, high-fat’ and ‘high-carbohydrate, low-fat’ diets found that the low-carbohydrate diet had a greater effect on weight loss than the high-carbohydrate diet, but no differences were observed for fat mass, glucose, and triglyceride levels, and blood pressure [41]. Results from prospective cohort studies, which investigated the effect of long-term dietary macronutrient distribution without calorie restriction, reported an association between low-carbohydrate intake and increased mortality [79, 80, 81]. Conversely, multinational and Asian studies have suggested that a high-carbohydrate intake contributed to increased mortality [82,83]. Interestingly, in a large prospective cohort study with a 25-year follow-up, midlife participants who had low (<40%) or high (>70%) energy from carbohydrate consumption were associated with increased mortality [84]. Moreover, those with a 50~55% carbohydrate intake showed the greatest lifespan, a level that might be considered moderate in the West but low in Asia. These conflicting results suggest the fact that the effects of macronutrient challenge in the short term, or energy restriction conditions might be different to those under long-term dietary intake and free-living individual conditions. 

Although our study analyzed multiple variants of circadian core clock genes in Korean population cohort data, there were some limitations. The SNPs from the genomic data of the cohort did not cover the full list of variants, resulting in missing SNPs reported in previous studies. Therefore, the analysis of comprehensive genetic variant data including crucial variants will provide additional important SNPs. Secondly, our study analyzed local community-based cohort data because of the availability of genomic data. To confirm these findings, futures studies based on a national representative cohort study with a larger sample size are required. Third, even though we included the covariates (i.e., age, BMI, and energy intake) for adjustment in a statistical analysis process, the possibility of effects induced by potential confounding factors, such as residential area, socioeconomic position, and health-related behaviors, should be considered.

In conclusion, we investigated Korean macronutrient intake patterns and found associations between the patterns and circadian clock gene variants, and obesity risk. The VLFC pattern was related to higher incidences of obesity and abdominal obesity in females. After the genotype stratification of nine SNPs of circadian genes, the association between the FC ratio and obesity risk differed by the genetic variants of *CLOCK*, *PER2*, and *CRY1*. These findings suggest that the low dietary FC ratio influences obesity susceptibility and the association depends on circadian clock genetic variations. Our findings highlight an important role of the association of macronutrient distribution and circadian clock on obesity.

## Figures and Tables

**Table 1:** The list of SNPs analyzed in this study.

Gene	SNP ID	Chromosome	Location	Alleles	MAF	HWE
*CLOCK*	rs11932595	4	55457430	A/G	0.1065	0.6955
	rs9312661	4	55476159	G/A	0.3604	0.2992
*ARNTL*	rs10766065	11	13256414	T/C	0.4983	0.9491
	rs9633835	11	13324046	A/G	0.4665	0.8643
*PER2*	rs2304672	2	238277948	G/C	0.0620	0.5825
	rs3741892	12	106993385	G/C	0.2321	0.4557
*CRY1*	rs11113192	12	107119148	G/C	0.2528	0.1215
	rs2541891	12	107184503	C/G	0.4131	0.3236
*CRY2*	rs7951225	11	45853841	A/T	0.3498	0.5747

MAF, minor allele frequency; HWE, Hardy–Weinberg equilibrium. Alleles are presented as major/minor allele.

**Table 2:** General characteristics and nutritional data by tertile of FC ratio.

Variables	Male					Female				
	VLFC (T1) (*n* = 918)	LFC (T2) (*n* = 919)	OFC (T3) (*n* = 919)	*p*	*Post Hoc*	VLFC (T1) (*n* = 862)	LFC (T2) (*n* = 863)	OFC (T3) (*n* = 862)	*p*	*Post Hoc*
General characteristics										
Age (year)	50.8 ± 7.3	48.6 ± 6.7	47.3 ± 6.4	<0.0001	A-B-C	53.2 ± 7.5	49.7 ± 7.5	46.9 ± 6.3	<0.0001	A-B-C
BMI (kg/m^2^)	24.2 ± 2.9	24.7 ± 2.9	24.6 ±2.8	0.0030	A-B-B	25.3 ± 3.4	24.7 ± 3.1	24.6 ± 3.3	<0.0001	A-B-B
Residential area—Urban	522 (56.9)	728 (79.3)	779 (84.8)	<0.0001	-	361 (41.9)	590 (68.4)	672 (78)	<0.0001	-
Body composition ^(1)^										
Lean body mass (kg)	52.7 ± 5.8	54.2 ±6.1	54.6 ± 6.0	<0.0001	A-B-B	39.8 ± 4.6	40.2 ± 4.3	40.7 ± 4.3	0.0011	A-B-B
Lean body mass (%)	78.1 ± 5.0	77.8 ± 4.9	78.2 ± 4.6	0.2600	A-B-B	67.7 ± 5.5	68.5 ± 4.9	68.9 ± 5.3	0.0002	A-B-B
Body fat (kg)	15.1 ± 4.7	15.7 ± 4.9	15.5 ± 4.6	0.0354	A-B-B	19.4 ± 5.3	18.8 ± 4.9	18.8 ± 5.3	0.0375	A-B-B
Body fat (%)	21.9 ± 4.9	22.1 ± 4.9	21.8 ± 4.5	0.3834	A-B-B	32.4 ± 5.1	31.5 ± 4.9	31.1 ± 5.2	<0.0001	A-B-B
Waist to hip ratio	0.90 ± 0.04	0.90 ± 0.04	0.90 ± 0.04	0.1779	A-B-B	0.91 ± 0.05	0.90 ± 0.05	0.89 ± 0.05	<0.0001	A-B-B
Nutritional intake										
Energy (kcal/day)	1766.0 ± 509.6	1979.1 ± 423.3	2250.2 ± 527.9	<0.0001	A-B-C	1640.4 ± 522.7	1834.6 ± 473.7	2020.5 ± 614.5	<0.0001	A-B-C
Carbohydrate (g/day)	333.4 ± 99.3	344.0 ± 74.4	350.8 ± 82.3	0.0002	A-B-B	320.8 ± 103.4	330.8 ± 87.8	325.5 ± 100.0	0.095	A-A-A
Protein (g/day)	53.5 ± 16.4	67.9 ± 16.4	87.3 ± 24.3	<0.0001	A-B-C	47.8 ± 16.4	61.8 ± 16.9	76.9 ± 25.7	<0.0001	A-B-C
Fat (g/day)	21.4 ± 7.7	34.6 ± 8.4	53.1 ± 16.2	<0.0001	A-B-C	15.9 ± 6.6	27.7 ± 8.0	44.2 ± 16.9	<0.0001	A-B-C
% Energy from each macronutrient										
Carbohydrate	75.5 ± 3.1	69.5± 1.9	62.5 ± 4.1	<0.0001	A-B-C	78.2 ± 3.0	72.1 ± 2.1	64.5 ± 4.5	<0.0001	A-B-C
Protein	12.1 ± 1.5	13.7 ± 1.4	15.5 ± 2.0	<0.0001	A-B-C	11.7± 1.4	13.5 ± 1.6	15.3 ± 2.0	<0.0001	A-B-C
Fat	10.8 ± 2.2	15.7 ± 1.2	21.1 ± 2.9	<0.0001	A-B-C	8.6 ± 2.0	13.5 ± 1.3	19.6 ± 3.5	<0.0001	A-B-C
FC ratio	0.14 ± 0.03	0.23 ± 0.02	0.34 ± 0.08	<0.0001	A-B-C	0.11 ± 0.03	0.19 ± 0.02	0.31 ± 0.09	<0.0001	A-B-C
Number of regular meal (meal/day)	2.9 ± 0.3	2.9 ± 0.3	2.8 ± 0.4	<0.0001	A-B-C	2.9 ± 0.3	2.8 ± 0.4	2.7 ± 0.5	<0.0001	A-B-C
Alcohol intake (g/day)	16.0 ± 24.6	18.0 ± 26.5	24.8 ± 32.7	<0.0001	A-A-B	1.0 ± 4.0	1.2 ± 4.2	2.4 ± 7.6	<0.0001	A-A-B
Tobacco consumption (pack/year)	17.9 ± 17.2	16.4 ± 16.2	18.2 ± 17.3	0.0585	A-A-A	0.3 ± 2.7	0.3 ± 2.9	0.4 ± 2.4	0.9189	A-A-A
Sleep duration (h)	6.9 ± 1.2	6.7 ± 1.3	6.6 ± 1.3	0.0003	A-B-B	6.8 ± 1.4	6.6 ± 1.4	6.4 ± 1.4	<0.0001	A-B-C
Moderate physical activity ^(2)^	314 (34.2)	322 (35.0)	392 (42.7)	0.0002	-	244 (28.3)	337 (39.0)	347 (40.3)	<0.0001	-

VLFC, Very low FC; LFC, Low FC; OFC, Optimal FC. Data are presented as mean ± standard deviation and number (percentage). ANOVA analysis with Tukey post hoc test and Welch’s ANOVA with Games–Howell test for adjusting unequal variances. ^(1)^ Data were collected from subjects who completed body composition analysis; male: *n* = 725, *n* = 819, *n* = 844; female: *n* = 641, *n* = 739, and *n* = 761. ^(2)^ ≥30 min per day.

**Table 3:** The association between tertiles of FC ratio and prevalence of disease.

	Male	*p*	Female	*p*
Obesity ^(1)^				
VLFC (T1)	1.15 (0.93–1.42)	0.205	1.50 (1.20–1.86)	0.000
LFC (T2)	1.29 (1.07–1.57)	0.010	1.12 (0.91–1.37)	0.281
OFC (T3)	1		1	
Abdominal obesity ^(2)^				
VLFC (T1)	0.92 (0.64–1.33)	0.670	1.84 (1.36–2.48)	<0.0001
LFC (T2)	0.87 (0.54–1.40)	0.449	0.90 (0.67–1.20)	0.462
OFC (T3)	1		1	

All odds ratios (OR) and 95% confidence intervals (CI) were calculated by performing multiple logistic regression. ^(1)^ BMI ≥25 kg/m^2^, odds ratio adjusted for age, sleep duration, energy intake, number of regular meals, alcohol intake, tobacco consumption, and moderate physical activity. ^(2)^ Waist circumference ≥90 cm for males and ≥85 cm for females, odds ratio adjusted for age, BMI, sleep duration, energy intake, number of regular meals, alcohol intake, tobacco consumption, and moderate physical activity.

**Table 4:** Prevalence of diseases by macronutrient intake patterns and genetic variants in males.

Gene	SNP		Obesity ^(1)^				Abdominal Obesity ^(2)^			
			VLFC (T1)	LFC (T2)	OFC (T3)	*p*- Interaction	VLFC (T1)	LFC (T2)	OFC (T3)	*p*- Interaction
*CLOCK*	rs11932595	AA	1.14 (0.90–1.44)	1.31 (1.06–1.63)	1	0.892	0.95 (0.63–1.43)	0.96 (0.66–1.39)	1	0.604
		GA/GG	1.41 (1.00–1.98)	1.46 (1.04–2.04)	1.21 (0.86–1.69)		1.18 (0.67–2.07)	0.93 (0.52–1.66)	1.45 (0.84–2.51)	
	rs9312661	AA	1.13 (0.83–1.54)	1.34 (0.99–1.80)	1	0.906	1.03 (0.59–1.77)	1.11 (0.67–1.84)	1	0.501
		GA/GG	1.35 (1.01–1.81)	1.47 (1.11–1.94)	1.16 (0.88–1.51)		1.11 (0.66–1.86)	0.95 (0.58–1.56)	1.27 (0.80–2.03)	
*ARNTL*	rs10766065	TT	1.16 (0.79–1.71)	1.38 (0.96–2.00)	1	0.910	1.38 (0.71–2.68)	0.77 (0.40–1.47)	1	0.145
		CT/CC	1.21 (0.88–1.66)	1.34 (0.98–1.81)	1.06 (0.78–1.43)		1.08 (0.61–1.90)	1.24 (0.72–2.13)	1.33 (0.79–2.26)	
	rs9633835	AA	1.24 (0.86–1.79)	1.40 (0.97–2.02)	1	0.839	0.74 (0.39–1.38)	0.79 (0.42–1.46)	1	0.700
		GA/GG	1.24 (0.90–1.70)	1.40 (1.03–1.89)	1.11 (0.83–1.50)		0.71 (0.41–1.22)	0.65 (0.39–1.08)	0.71 (0.43–1.17)	
*PER2*	rs2304672	GG	1.18 (0.94–1.47)	1.33 (1.09–1.64)	1	0.665	1.01 (0.69–1.49)	0.89 (0.62–1.28)	1	0.281
		CG/CC	1.04 (0.69–1.59)	1.15 (0.78–1.70)	1.11 (0.72–1.70)		0.57 (0.27–1.21)	0.96 (0.50–1.86)	1.21 (0.60–2.44)	
*CRY1*	rs3741892	GG	1.01 (0.77–1.31)	1.14 (0.89–1.47)	1	0.180	0.75 (0.48–1.19)	0.74 (0.48–1.13)	1	0.262
		CG/CC	1.12 (0.84–1.50)	1.24 (0.95–1.63)	0.81 (0.62–1.05)		0.76 (0.46–1.25)	0.69 (0.44–1.10)	0.60 (0.38–0.96)	
	rs11113192	GG	1.50 (1.14–1.97)	1.52 (1.17–1.96)	1	0.009	1.10 (0.68–1.77)	1.02 (0.65–1.60)	1	0.542
		CG/CC	1.06 (0.80–1.41)	1.37 (1.04–1.80)	1.28 (0.98–1.67)		1.17 (0.70–1.95)	1.14 (0.71–1.83)	1.50 (0.95–2.36)	
	rs2541891	CC	1.31 (0.94–1.84)	1.47 (1.06–2.03)	1	0.522	0.77 (0.43–1.38)	1.01 (0.58–1.75)	1	0.385
		GC/GG	1.09 (0.81–1.47)	1.23 (0.93–1.64)	1.02 (0.77–1.35)		1.18 (0.71–1.96)	0.95 (0.58–1.55)	1.16 (0.72–1.88)	
*CRY2*	rs7951225	AA	1.17 (0.86–1.60)	1.44 (1.07–1.92)	1	0.617	0.94 (0.55–1.61)	1.20 (0.73–1.96)	1	0.159
		TA/TT	1.35 (1.01–1.80)	1.44 (1.09–1.89)	1.20 (0.91–1.56)		0.92 (0.56–1.50)	0.68 (0.42–1.11)	1.01 (0.64–1.60)	

All odds ratios and 95% confidence intervals were calculated by performing multiple logistic regression. *p*-interaction: interaction between SNP and FC tertiles. Data in **bold** indicate statistically significant value after Bonferroni correction for multiple comparisons (corrected *p*-value: 0.05/45 = 0.001). ^(1)^ BMI ≥ 25 kg/m^2^, odds ratio adjusted for age, sleep duration, energy intake, number of regular meals, alcohol intake, tobacco consumption, and moderate physical activity. ^(2)^ Waist circumference ≥90 cm for males, odds ratio adjusted for BMI and the same covariates as obesity.

**Table 5:** Prevalence of diseases by macronutrient intake patterns and genetic variants in females.

Gene	SNP		Obesity ^(1)^				Abdominal Obesity ^(2)^			
			VLFC (T1)	LFC (T2)	OFC (T3)	*p*- Interaction	VLFC (T1)	LFC (T2)	OFC (T3)	*p*- Interaction
*CLOCK*	rs11932595	AA	1.35 (1.06–1.71)	1.04 (0.83–1.30)	1	0.093	**1.84 (1.32–2.56)**	0.84 (0.60–1.17)	1	0.572
		GA/GG	1.74 (1.23–2.46)	1.14 (0.81–1.61)	0.76 (0.53–1.08)		2.05 (1.30–3.22)	1.29 (0.79–2.09)	1.14 (0.68–1.93)	
	rs9312661	AA	1.17 (0.85–1.61)	0.95 (0.69–1.30)	1	0.123	**2.26 (1.43–3.56)**	0.93 (0.58–1.48)	1	0.404
		GA/GG	1.54 (1.14–2.07)	1.09 (0.82–1.45)	0.88 (0.66–1.16)		**2.11 (1.38–3.23)**	1.15 (0.75–1.77)	1.32 (0.86–2.02)	
*ARNTL*	rs10766065	TT	1.66 (1.11–2.47)	0.99 (0.67–1.47)		0.434	2.46 (1.39–4.35)	0.72 (0.39–1.34)	1	0.113
		CT/CC	1.56 (1.12–2.17)	1.25 (0.91–1.73)	1.08 (0.79–1.49)		2.15 (1.32–3.51)	1.22 (0.75–1.99)	1.30 (0.79–2.11)	
	rs9633835	AA	1.48 (0.99–2.13)	1.14 (0.79–1.65)	1	0.953	1.11 (0.66–1.87)	0.68 (0.39–1.16)	1	0.070
		GA/GG	1.64 (1.19–2.27)	1.20 (0.88–1.68)	1.09 (0.80–1.48)		1.56 (0.99–2.48)	0.70 (0.44–1.10)	0.7 (0.44–1.12)	
*PER2*	rs2304672	GG	**1.49 (1.18–1.87)**	1.14 (0.92–1.42)	1	0.670	**1.85 (1.35–2.54)**	0.87 (0.64–1.19)	1	0.827
		CG/CC	1.59 (1.04–2.43)	0.94 (0.60–1.47)	1.02 (0.66–1.56)		1.28 (0.74–2.22)	0.76 (0.39–1.49)	0.68 (0.34–1.34)	
*CRY1*	rs3741892	GG	**1.60 (1.22–2.10)**	1.16 (0.90–1.51)	1	0.728	**1.90 (1.30–2.76)**	0.86 (0.59–1.26)	1	0.793
		CG/CC	**1.76 (1.30–2.38)**	1.38 (1.03–1.83)	1.29 (0.98–1.71)		1.70 (1.13–2.56)	0.93 (0.61–1.41)	0.98 (0.64–1.49)	
	rs11113192	GG	1.48 (1.12–1.96)	1.14 (0.87–1.49)	1	0.960	1.84 (1.25–2.70)	0.91 (0.61–1.34)	1	0.995
		CG/CC	1.37 (1.02–1.84)	1.00 (0.75–1.32)	0.91 (0.69–1.20)		1.89 (1.26–2.82)	0.91 (0.60–1.37)	1.03 (0.68–1.56)	
	rs2541891	CC	1.36 (0.96–1.94)	0.91 (0.64–1.28)	1	0.341	1.96 (1.20–3.20)	0.80 (0.48–1.32)	1	0.621
		GC/GG	1.55 (1.13–2.11)	1.23 (0.91–1.65)	0.99 (0.74–1.33)		1.96 (1.26–3.06)	1.05 (0.67–1.64)	1.11 (0.71–1.72)	
*CRY2*	rs7951225	AA	1.52 (1.10–2.08)	1.10 (0.80–1.51)	1	0.981	2.07 (1.32–3.24)	0.93 (0.58–1.49)	1	0.768
		TA/TT	1.63 (1.20–2.23)	1.23 (0.92–1.64)	1.09 (0.82–1.45)		1.96 (1.26–3.02)	1.01 (0.66–1.56)	1.15 (0.75–1.77)	

All odds ratios and 95% confidence intervals were calculated by performing multiple logistic regression. *p*-interaction: interaction between SNP and FC tertiles. Data in **bold** indicate statistically significant value after Bonferroni correction for multiple comparisons (corrected *p*-value: 0.05/45 = 0.001). ^(1)^ BMI ≥ 25 kg/m^2^, odds ratio adjusted for age, sleep duration, energy intake, number of regular meals, alcohol intake, tobacco consumption, and moderate physical activity. ^(2)^ Waist circumference ≥85 cm for females, odds ratio adjusted for BMI and the same covariates as obesity.

**Figure A1:**
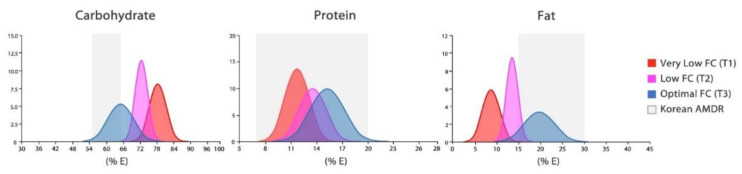
Macronutrient distribution according to FC group in females. Each macronutrient distribution of three FC groups was presented. The Very Low FC is represented in red; the Low FC and Optimal FC are represented in purple and blue, respectively. The gray background indicates Korean acceptable macronutrient distribution range (AMDR).

## Data Availability

The KoGES data are available on request from the National Research Institute of Health [47].

## Appendix A

**Table A1:** The expression quantitative trait loci information of SNPs.

SNP ID	Gene Symbol	Alleles	*p*-Value	Tissue
rs11932595	*CLOCK*	A/G	3 × 10^−6^	Muscle–Skeletal
			5 × 10^−7^	Cells–Cultured fibroblasts
rs9312661	*CLOCK*	A/G	2 × 10^−25^	Thyroid
			2 × 10^−17^	Skin–Sun Exposed (Lower leg)
			9 × 10^−17^	Skin–Not Sun Exposed (Suprapubic)
			3 × 10^−14^	Lung
			5 × 10^−14^	Nerve–Tibial
			8 × 10^−14^	Cells–Cultured fibroblasts
			7 × 10^−13^	Spleen
			7 × 10^−13^	Testis
			6 × 10^−10^	Small Intestine–Terminal Ileum
			7 × 10^−10^	Esophagus–Mucosa
			1 × 10^−9^	Pancreas
			7 × 10^−9^	Artery–Aorta
			2 × 10^−7^	Colon–Transverse
			2 × 10^−6^	Whole Blood
			1 × 10^−5^	Breast–Mammary Tissue
			2 × 10^−5^	Esophagus–Gastroesophageal Junction
			3 × 10^−5^	Artery–Tibial
			1 × 10^−4^	Adipose–Subcutaneous
rs10766065	*ARNTL*	T/C	1 × 10^−12^	Whole Blood
rs9633835	*ARNTL*	A/G	1 × 10^−16^	Whole Blood
rs2304672	*PER2*	G/C	2 × 10^−6^	Thyroid
rs3741892	*CRY1*	G/C	1 × 10^−35^	Testis
			4 × 10^−10^	Muscle–Skeletal
rs11113192	*CRY1*	G/C	1 × 10^−6^	Testis
			6 × 10^−5^	Esophagus–Gastroesophageal Junction
rs2541891	*CRY1*	C/G	1 × 10^−4^	Testis
rs7951225	*CRY2*	A/T	3 × 10^−12^	Whole Blood
			1 × 10^−5^	Artery–Aorta
			1 × 10^−5^	Artery–Tibial
			3 × 10^−5^	Spleen

Expression quantitative loci (eQTL) information from GTEx database. Alleles are presented as major/minor allele.

## Appendix B

**Table A2:** General characteristics of subjects.

Variables	Total (*n* = 5343)			Male (*n* = 2756)			Female (*n* = 2587)		
General characteristics									
Age (year)	49.4 ± 7.3			48.9 ± 7.0			49.9 ± 7.6		
BMI (kg/m^2^)	24.7 ± 3.1			24.5 ± 2.9			24.8 ± 3.2		
Sleep duration (h)	6.7 ± 1.3			6.7 ± 1.3			6.6 ± 1.4		
Nutritional intake									
Energy (kcal/day)	1917.8 ± 550.6			1998.5 ± 527.5			1831.8 ± 561.8		
Carbohydrate (g/day)	334.5 ± 92.2			342.7 ± 86.2			325.7 ± 97.3		
Protein (g/day)	66.0 ± 23.9			69.6 ± 23.8			62.2 ± 23.4		
Fat (g/day)	32.9 ± 17.2			36.4 ± 17.3			29.3 ± 16.3		
% Energy from each macronutrient									
Protein	13.6 ± 2.2			13.8 ± 2.2			13.5 ± 2.2		
Carbohydrate	70.3 ± 6.5			69.2 ± 6.2			71.6 ± 6.6		
Fat	14.9 ± 5.0			15.9 ± 4.7			13.9 ± 5.1		
Number of regular meal	2.8 ± 0.4			2.9 ± 0.3			2.8 ± 0.4		
Alcohol intake (g/day)	10.8 ± 22.6			19.6 ± 28.4			1.5 ± 5.5		
Tobacco consumption (pack/year)	9.2 ± 15.0			17.5 ± 16.9			0.3 ± 2.7		
Moderate physical activity ^(1)^	1956 (36.7)			1028 (37.3)			928 (35.87)		

Data are presented as the mean ± standard deviation and number (percentage). ^(^^1)^ ≥30 min per day.
